# Longitudinal Changes in Lipid Profile After Sustained Virological Response in Patients with Chronic Hepatitis C Treated with Direct-Acting Antivirals

**DOI:** 10.3390/healthcare14040486

**Published:** 2026-02-14

**Authors:** Oana Koppandi, Bogdan Miutescu, Iulia Ratiu, Alexandru Popa, Camelia Nica, Eyad Gadour, Bogdan Dan Totolici, Raluca Lupusoru, Ana Maria Ghiuchici, Eftimie Miutescu

**Affiliations:** 1 Department of Gastroenterology, Faculty of Medicine, Western University “Vasile Goldis” of Arad, Revolutiei Boulevard 94, 310025 Arad, Romania; koppandi.oana@uvvg.ro (O.K.);; 2 Multidisciplinary Doctoral School, Western University “Vasile Goldis” of Arad, Revolutiei Boulevard 94, 310025 Arad, Romania; 3 Department of Gastroenterology and Hepatology, Faculty of Medicine, “Victor Babes” University of Medicine and Pharmacy Timisoara, Eftimie Murgu Square 2, 300041 Timisoara, Romania; 4 Advanced Regional Research Center in Gastroenterology and Hepatology, “Victor Babes” University of Medicine and Pharmacy Timisoara, Eftimie Murgu Square 2, 300041 Timisoara, Romania; 5 Department of Gastroenterology and Hepatology, King Abdulaziz Hospital-National Guard, Ahsa 31982, Saudi Arabia; 6 Department of Internal Medicine, Faculty of Medicine, Zamzam University College, Khartoum 11113, Sudan; 7 Department of Surgery, Faculty of Medicine, Western University “Vasile Goldis” of Arad, Revolutiei Boulevard 94, 310025 Arad, Romania; 8 Department III, Functional Science, Discipline of Medical Informatics and Biostatistics, “Victor Babes” University of Medicine and Pharmacy Timisoara, Eftimie Murgu Square 2, 300041 Timisoara, Romania; 9 Center for Modeling Biological Systems and Data Analysis, “Victor Babes” University of Medicine and Pharmacy Timisoara, Eftimie Murgu Square 2, 300041 Timisoara, Romania

**Keywords:** chronic hepatitis C, direct-acting antivirals, sustained virological response, lipid metabolism, long-term follow-up, low-density lipoprotein cholesterol, cardiovascular risk

## Abstract

**Highlights:**

**What are the main findings?**

- Long-term follow-up after HCV cure revealed sustained increases in total cholesterol and LDL-C, with stabilization over time.
- Lipid changes after SVR occurred independently of major changes in body mass index and were consistent across fibrosis stages.

**What are the implications of the main findings?**

- Metabolic monitoring should be considered an integral part of post-SVR follow-up in patients cured of chronic hepatitis C.
- Awareness of post-cure lipid changes may help optimize cardiovascular risk assessment in the growing population of HCV-cured patients.

**Abstract:**

**Background:** Direct-acting antiviral (DAA) therapy has transformed chronic hepatitis C virus (HCV) infection into a curable disease. Beyond viral eradication, increasing attention has been directed toward metabolic changes following sustained virological response (SVR), particularly alterations in lipid metabolism. This study aimed to assess the long-term evolution of lipid parameters after HCV cure in a real-world clinical cohort. **Methods:** We conducted a prospective, single-center observational study including 85 patients with chronic HCV infection who achieved SVR after DAA therapy. Lipid parameters, including total cholesterol, low-density lipoprotein cholesterol (LDL-C), high-density lipoprotein cholesterol (HDL-C), and triglycerides, were assessed at baseline and during post-SVR follow-up at 24, 48, and 96 weeks. Body mass index (BMI) and non-invasive fibrosis indices were also evaluated. Longitudinal changes were analyzed using mixed-effects models. **Results:** Total cholesterol increased from 157.7 ± 35.6 mg/dL at baseline to 179.6 ± 42.9 mg/dL at SVR 24 and further to 189.0 ± 40.3 mg/dL at SVR 48, stabilizing at 177.7 ± 38.3 mg/dL at SVR 96. LDL-C showed a similar trajectory from 94.6 ± 30.8 mg/dL at baseline to 107.5 ± 33.3 mg/dL at SVR 24, further raising to 115.7 ± 36.2 mg/dL at SVR48, and 111.8 ± 39.5 mg/dL at SVR 96. HDL-C showed minimal change, while triglycerides demonstrated greater interindividual variability without a consistent population-level trend. BMI remained stable over follow-up (26.6 ± 4.7 to 27.6 kg/m^2^). Linear mixed-effects models confirmed a significant effect of time after SVR on total cholesterol and LDL-C (*p* < 0.05). **Conclusions:** In this real-world cohort, HCV cure with DAA therapy was associated with sustained long-term changes in lipid metabolism, characterized by increases in total cholesterol and LDL-C independent of major weight changes. These findings support the importance of continued metabolic monitoring after SVR, particularly in patients with additional cardiometabolic risk factors.

## 1. Introduction

Chronic hepatitis C virus (HCV) infection remains a relevant public health and clinical concern, despite major advances in antiviral therapy over the past decade. The burden of disease is unevenly distributed worldwide, with the highest prevalence reported in the Eastern Mediterranean Region, where an estimated 12 million individuals are chronically infected [1]. The introduction of direct-acting antivirals (DAAs) has dramatically improved treatment efficacy, allowing sustained virological response (SVR) to be achieved in most patients and effectively transforming HCV into a curable disease [2,3]. As a result, morbidity and mortality related to progressive liver disease have declined substantially [4]. However, growing clinical evidence suggests that viral eradication does not necessarily represent the end of disease-related follow-up, as patients may continue to experience systemic and metabolic alterations after cure [5,6].

Chronic HCV infection is associated with clinically relevant alterations in lipid metabolism, mostly reflected by reduced serum cholesterol and low-density lipoprotein cholesterol (LDL-C) levels in untreated patients. These changes are thought to be related to virus-induced interference with hepatic lipid handling and are frequently observed in routine clinical practice [7,8]. Following successful treatment with DAAs and achievement of SVR, multiple studies have consistently reported increases in total cholesterol and LDL-C during early post-treatment follow-up, suggesting a reversal of HCV-related metabolic alterations [5,9,10,11,12]. Recent real-world evidence indicates that these lipid changes may persist beyond the immediate post-SVR period, underscoring the need for continued metabolic surveillance after viral eradication [7]. As a result, lipid profile assessment has become an increasingly relevant component of post-cure clinical monitoring in patients with chronic hepatitis C.

Despite increasing recognition of post-treatment metabolic changes in patients cured of HCV, the available evidence remains largely limited to short-term follow-up. Most published studies have evaluated lipid parameters at 12 or 24 weeks after achieving SVR, providing important insights into early metabolic shifts following viral eradication [5,13]. However, data extending beyond the first post-SVR year are scarce, particularly from real-world clinical cohorts [13,14]. As a result, it remains unclear whether post-SVR lipid alterations persist, stabilize, or evolve over time during longer-term follow-up. This lack of longitudinal data represents an important gap in current knowledge, with potential implications for post-cure monitoring strategies and long-term metabolic risk assessment in patients with chronic hepatitis C.

The aim of the present study was to evaluate the long-term evolution of lipid parameters in patients with chronic HCV infection that achieved SVR after treatment with DAAs. Using a real-world longitudinal cohort with repeated post-SVR assessments, we sought to characterize temporal patterns of lipid changes during extended follow-up. By providing data beyond the early post-treatment period, this study aims to contribute to a better understanding of metabolic changes after HCV cure and to inform post-SVR clinical monitoring strategies.

## 2. Materials and Methods

We conducted a prospective, single-center observational longitudinal study at Arad County Emergency Clinical Hospital, Arad, Romania. A total of 85 consecutive outpatients diagnosed with chronic HCV infection who initiated treatment with DAA regimens between July 2022 and December 2024 were considered for inclusion. The study was designed to evaluate long-term metabolic changes SVR under real-world clinical conditions. Adult patients (≥18 years) with confirmed chronic HCV infection who achieved SVR after DAA therapy were eligible for inclusion. SVR was defined as undetectable HCV RNA at 12 weeks after completion of antiviral treatment. Patients were enrolled consecutively during routine outpatient follow-up visits. Exclusion criteria included failure to achieve SVR, decompensated liver disease, co-infection with human immunodeficiency virus (HIV), active hepatocellular carcinoma (HCC), and incomplete clinical or laboratory follow-up data. Patients with decompensated cirrhosis were not included, as such cases are routinely managed in tertiary hepatology centers. Liver cirrhosis was diagnosed based on a combination of clinical assessment, laboratory findings, and imaging or endoscopic evaluation, supported by non-invasive methods such as FibroMax^®^ (BioPredictive, Paris, France) and FibroScan^®^ (Echosens, Paris, France) (≥12.5 kPa), when available. For subgroup analyses, baseline liver fibrosis stage was grouped into no or mild fibrosis (F0–F2) and advanced fibrosis (F3–F4), a classification commonly used in clinical practice to distinguish patients at higher risk of liver-related complications.

Demographic and clinical data, including age, sex, body mass index (BMI), liver disease severity, comorbidities, and antiviral treatment regimen, were collected from medical records at baseline. Patients were followed longitudinally with laboratory and clinical parameters assessed at baseline and during post-SVR follow-up visits performed at 24 weeks (SVR 24), 48 weeks (SVR 48), and up to 96 weeks (SVR 96) after achieving SVR, depending on clinical availability. Post-SVR follow-up time points are reported relative to the date of SVR confirmation.

Blood samples were obtained after an overnight fast and analyzed in the hospital’s certified central laboratory according to routine clinical practice and institutional quality-control procedures. The lipid profile included total cholesterol, LDL-C, high-density lipoprotein cholesterol (HDL-C), and triglycerides, measured using standardized enzymatic colorimetric assays. Additional biochemical parameters, including liver enzymes and platelet counts, were assessed using automated laboratory methods. Non-invasive fibrosis indices—the fibrosis-4 (FIB-4) index and the aspartate aminotransferase-to-platelet ratio index (APRI)—were calculated using validated formulas originally described by Sterling et al. [15] and Wai et al. [16] derived from routinely collected laboratory variables. Cardiometabolic comorbidities were identified from medical records and included type 2 diabetes mellitus, essential arterial hypertension, dyslipidemia, ischemic heart disease, heart failure, atrial fibrillation, prior cerebrovascular events, chronic kidney disease and chronic obstructive pulmonary disease. Patients were classified according to the presence or absence of at least one cardiometabolic comorbidity. This grouping was chosen based on the established association of these conditions with metabolic dysfunction and cardiovascular risk and to allow clinically meaningful subgroup analyses in a real-world cohort.

The primary outcome was the longitudinal evolution of lipid parameters after viral eradication. Secondary outcomes included changes in BMI and non-invasive fibrosis indices during follow-up.

Generative artificial intelligence tools were used to assist with language editing and manuscript drafting. These tools were not used for data generation, statistical analysis, or interpretation of study results. All scientific content was reviewed and validated by the authors.

### Statistical Analysis

Continuous variables were assessed for normality using visual inspection and distribution-based methods and are presented as means ± standard deviation or medians with interquartile ranges, as appropriate. Categorical variables are summarized as counts and percentages.

Longitudinal changes in lipid parameters were analyzed using linear mixed-effects models, with time after SVR included as a fixed effect and patient identity included as a random effect to account for repeated measurements within individuals. This approach allowed inclusion of all available observations and appropriately accounted for unbalanced data resulting from incomplete follow-up, thereby reducing the risk of attrition-related bias. Secondary descriptive analyses were performed to explore lipid trajectories according to baseline fibrosis stage and cardiometabolic comorbidity status. No formal hypothesis testing was conducted for these subgroup analyses.

Sensitivity analyses were performed by repeating the primary longitudinal lipid analyses after excluding patients with advanced fibrosis (F3–F4) to assess the robustness of the findings.

All statistical analyses were performed using Python (version 3.x; Python Software Foundation, Wilmington, DE, USA) with the pandas, NumPy, SciPy, and statsmodels packages, and statistical significance was defined as a two-sided *p*-value < 0.05. Because this was a real-world observational study, no formal a priori sample size calculation was performed; all eligible patients treated during the study period were consecutively included.

## 3. Results

### 3.1. Baseline Characteristics

A total of 85 patients with chronic hepatitis C who achieved SVR after DAA therapy were included in the analysis. The mean age of the study population was 61.9 years, and 64.7% were female. At baseline, the mean BMI was 26.57 kg/m^2^. Liver cirrhosis (F4 fibrosis) was present in 36.5% of the patients.

Cardiometabolic comorbidities were identified in 55 patients, while 14 patients had at least one other chronic comorbidity. Baseline lipid parameters, non-invasive fibrosis indices and antiviral regimens are summarized in Table 1.

A comparison of baseline characteristics between patients with available SVR96 follow-up and those without extended follow-up demonstrated broadly similar demographic and clinical profiles (Table 2). Although cardiometabolic comorbidities were more frequent among patients without SVR96 data, no clinically meaningful differences were observed in baseline lipid parameters, suggesting a limited risk of significant selection bias.

### 3.2. Follow-Up Availability

**Figure 1:**
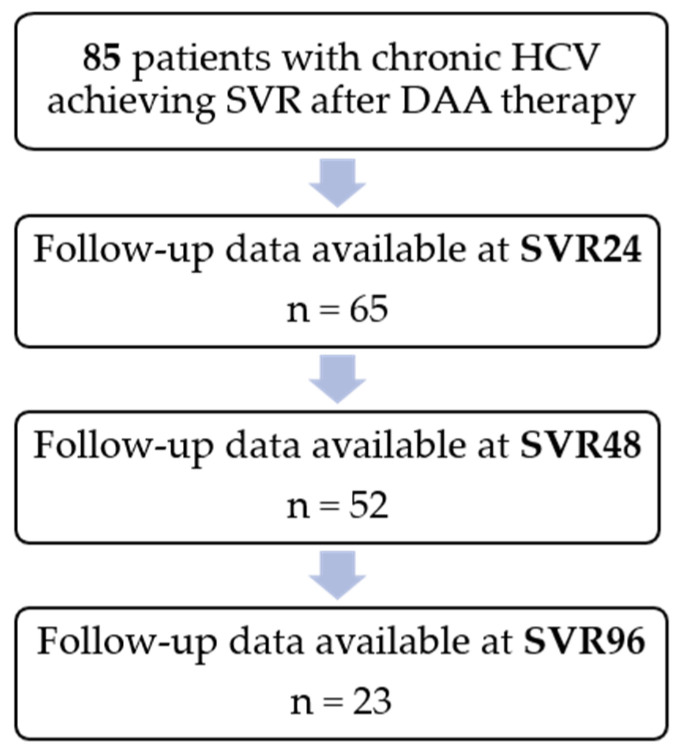
Flow diagram of patient inclusion and follow-up availability across post-SVR time points. Decreasing sample size reflects routine real-world clinical follow-up.

Post-SVR follow-up data were available for 65 patients at SVR 24, 52 patients at SVR 48, and 23 patients at SVR 96. Attrition over time reflects the real-world design of the cohort, in which long-term laboratory follow-up was not consistently available for all patients. (Figure 1).

### 3.3. Longitudinal Changes in Lipid Parameters

Longitudinal analysis demonstrated significant changes in lipid parameters following SVR. Mean total cholesterol increased by approximately 22 mg/dL from baseline (157.7 ± 35.6 mg/dL) to 179.6 ± 42.9 mg/dL at SVR 24 and peaked at SVR 48 (189.0 ± 40.3 mg/dL) to 177.7 ± 38.3 mg/dL during extended follow-up (SVR 96).

**Figure 2:**
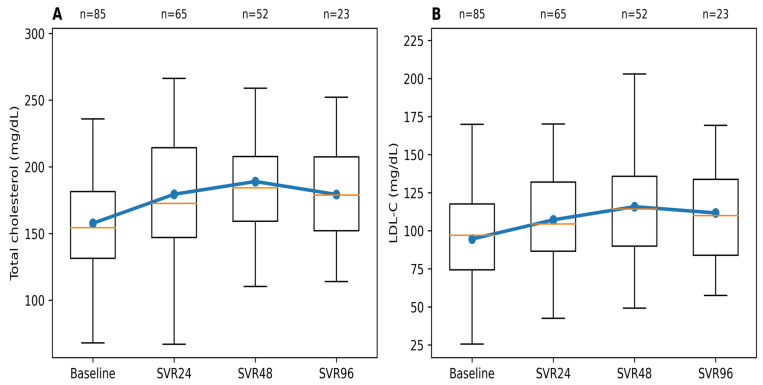
Longitudinal changes in total cholesterol and LDL-C following SVR. (**A**) Total cholesterol and (**B**) LDL-C values at baseline and during post-treatment follow-up. Boxplots represent the distribution at each time point (median and interquartile range), with whiskers indicating the full range of observed values. The blue line and markers denote mean values across time points, while the orange line within each box indicates the median. The number of patients with available data at each time point is shown above the plots.

A similar pattern was observed for LDL-C, which increased by a mean of 13 mg/dL at baseline (94.6 ± 30.8 mg/dL) to 107.5 ± 33.3 mg/dL at SVR 24 and 115.7 ± 36.2 mg/dL at SVR 48, followed by 111.8 ± 39.5 mg/dL at SVR 96 at the extended follow-up period, a change that may warrant closer cardiovascular risk monitoring following viral eradication. (Figure 2).

These trends were confirmed using linear mixed-effects models accounting for repeated measurements and incomplete follow-up, demonstrating a significant overall effect of time after SVR on both total cholesterol and LDL-C.

#### Longitudinal Changes in HDL Cholesterol and Triglyceride Levels

**Figure 3:**
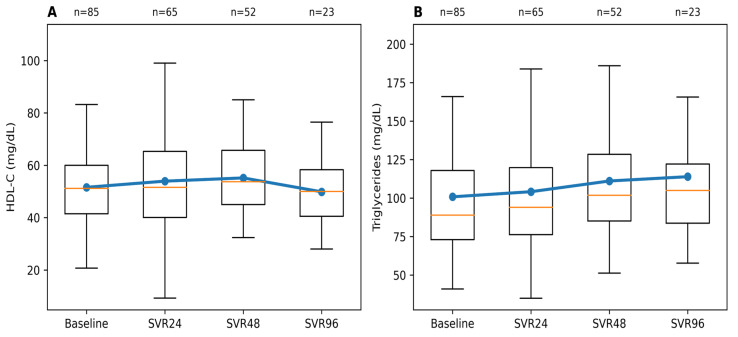
Longitudinal changes in HDL-C and triglycerides following SVR. (**A**) HDL-C and (**B**) triglyceride levels at baseline and during post-treatment follow-up. Boxplots represent the distribution at each time point (median and interquartile range), with whiskers indicating the full range of observed values. The blue line and markers denote mean values across time points, while the orange line within each box indicates the median. The number of patients with available data at each time point is shown above the plots.

HDL-C demonstrated modest changes following SVR. Mean HDL-C levels increased by approximately 3–4 mg/dL (51.6 ± 13.9 mg/dL) from baseline to 54.2 ± 17.8 mg/dL at SVR 24 and 55.4 ± 12.5 mg/dL at SVR 48. At extended follow-up, mean HDL-C was 49.6 ± 13.5 mg/dL (SVR 96). Overall, HDL-C showed a mild early increase after viral eradication, followed by stabilization during longer-term follow-up. Longitudinal HDL-C trends are illustrated in Figure 3A. Triglyceride levels exhibited greater interindividual variability compared with cholesterol fractions. Median triglyceride values increased from 89 mg/dL (IQR 73–118) at baseline to 94 mg/dL (IQR 76–121) at SVR 24 and 102 mg/dL (IQR 85–126) at SVR 48. At extended follow-up (SVR 96), median triglyceride levels were 110 mg/dL (IQR 82–124). Although a gradual increase in median values was observed, no clearly defined longitudinal pattern was evident at the population level. This variability may reflect differences in lifestyle factors, underlying metabolic characteristics, or genetic predisposition among patients.

Triglyceride trajectories are shown in Figure 3B.

### 3.4. Longitudinal Changes in BMI

**Figure 4:**
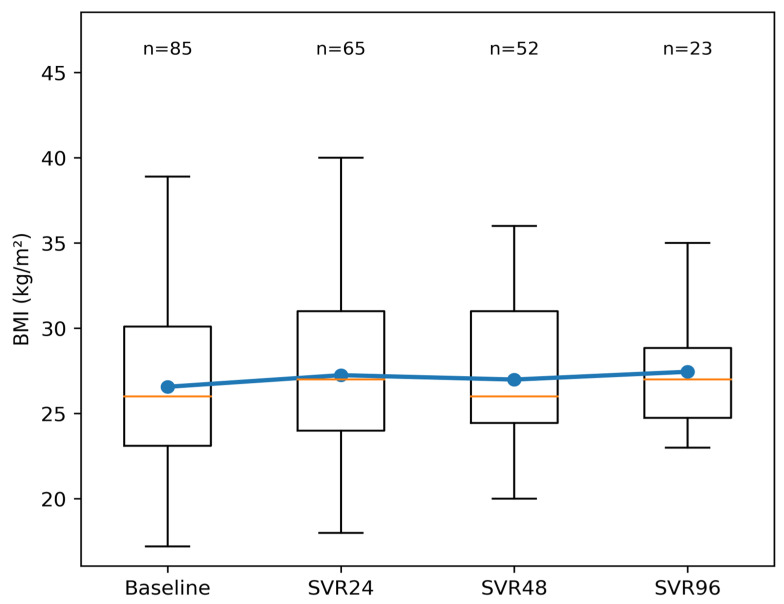
Longitudinal trajectory of BMI before treatment and during follow-up after SVR. Boxplots represent the distribution of BMI at each time point (median and interquartile range), with whiskers indicating the full range of observed values. The blue line and markers denote mean BMI values across time points, while the orange line within each box indicates the median. The number of patients with available data at each time point is shown above the plots.

BMI showed a modest increase during post-SVR follow-up. Mean BMI increased by approximately 1 kg/m^2^ from baseline (26.57 kg/m^2^)to 27.20 kg/m^2^ at SVR 24 and 27.52 kg/m^2^ at SVR 48, with similar values observed at extended follow-up (27.60 kg/m^2^ at SVR 96). Longitudinal analysis did not indicate abrupt or clinically meaningful weight changes over time. The observed BMI trajectory suggests that post-SVR lipid changes occurred largely independently of major variations in body weight suggesting a metabolic effect of viral clearance rather than secondary changes driven by body composition. BMI trajectories are illustrated in Figure 4.

### 3.5. Longitudinal Changes in Non-Invasive Fibrosis Indices

Non-invasive fibrosis indices demonstrated a consistent improvement following SVR. Both APRI and FIB-4 values declined markedly after viral eradication and remained lower throughout extended follow-up. The most pronounced decrease was observed within the first post-SVR assessment, followed by stabilization over time. Detailed longitudinal values for APRI and FIB-4 across follow-up time points are presented in Table 3.

These findings likely reflect reductions in hepatic inflammation and biochemical activity after HCV cure rather than structural fibrosis regression and are consistent with the expected post-SVR behavior of non-invasive fibrosis markers.

### 3.6. Lipid Trajectories According to Baseline Fibrosis Stage

When stratified by baseline fibrosis stage, patients with advanced fibrosis (F3–F4) exhibited lower baseline total cholesterol, LDL-C, and HDL-C levels compared with patients with no or mild fibrosis (F0–F2). Following SVR, lipid levels increased in both fibrosis groups. Total cholesterol and LDL-C rose during early post-SVR follow-up and subsequently stabilized during extended follow-up, with similar temporal patterns observed in both groups.

Despite these parallel trends, absolute lipid levels remained slightly lower in patients with advanced fibrosis across most follow-up time points. HDL-C showed modest changes over time in both groups, while triglyceride levels displayed greater variability without a clearly defined longitudinal pattern. Detailed lipid values according to fibrosis stage and follow-up time point are presented in Table 4.

### 3.7. Lipid Trajectories According to Cardiometabolic Comorbidity Status

When stratified by cardiometabolic comorbidity status, patients with and without cardiometabolic conditions showed similar temporal patterns of lipid changes after SVR. In both subgroups, total cholesterol and LDL-C increased after viral eradication and subsequently stabilized during longer-term follow-up. Differences between groups were primarily observed in absolute lipid levels rather than in the direction of change over time. Descriptive subgroup lipid values across follow-up time points are presented in Table 5.

### 3.8. Sensitivity Analysis Excluding Patients with Advanced Fibrosis

To assess the robustness of the observed lipid trajectories, a sensitivity analysis was performed after excluding patients with advanced fibrosis (F3–F4). In this restricted cohort, longitudinal changes in total cholesterol and LDL-C remained consistent with those observed in the full study population. Specifically, both lipid parameters increased after SVR and subsequently stabilized during extended follow-up.

The magnitude and temporal pattern of lipid changes were comparable to those seen in the primary analysis, indicating that the observed post-SVR lipid evolution was not driven exclusively by patients with advanced fibrosis.

Taken together, these findings underscore the importance of metabolic monitoring after SVR, particularly given the sustained increases observed in atherogenic lipid fractions.

## 4. Discussion

### 4.1. Overview of the Main Findings

In this real-world longitudinal study, we explored the long-term metabolic consequences of HCV eradication in patients treated with DAAs. Our findings indicate that lipid metabolism undergoes sustained changes after SVR, characterized by early post-treatment shifts followed by stabilization during extended follow-up. The consistency of these patterns across fibrosis stages and in the absence of major weight changes suggests that viral clearance itself plays a central role in shaping post-cure metabolic profiles.

### 4.2. Interpretation Changes After HCV Cure

The observed post-SVR increase in total cholesterol and LDL-C is consistent with previous studies describing early metabolic shifts after HCV eradication [17,18,19]. Chronic HCV infection interferes with hepatic lipid metabolism through virus–host interactions involving lipid droplets and lipoprotein assembly, leading to lower circulating cholesterol levels in untreated patients. Clearance of the virus appears to restore normal hepatic lipid handling, resulting in a rebound increase in cholesterol fractions [20,21]. This trajectory reflects the well-described “lipid paradox” in chronic hepatitis C, whereby active infection is associated with relatively lower circulating cholesterol levels that subsequently rise following viral eradication. By extending follow-up beyond the first post-SVR year, our findings demonstrate that these changes are not transient but persist over time, suggesting a sustained metabolic shift after viral clearance. In contrast to cholesterol fractions, HDL-C showed only modest changes during follow-up, while triglyceride levels displayed substantial interindividual variability without a consistent population-level trend. These findings align with existing evidence indicating that post-SVR metabolic changes predominantly affect cholesterol metabolism, whereas triglyceride levels may be more strongly influenced by individual cardiometabolic factors and lifestyle-related determinants [22]. Importantly, BMI remained largely stable throughout follow-up, indicating that lipid changes after SVR are not primarily driven by weight gain but may reflect intrinsic metabolic consequences of viral eradication [9].

The decline in non-invasive fibrosis indices observed after SVR in the present study most likely reflects improvements in hepatic inflammation and biochemical activity rather than true structural fibrosis regression. Chronic HCV infection is known to disrupt hepatic lipid handling and inflammatory pathways through virus–host interactions involving lipid droplets and lipoprotein assembly, and viral clearance leads to normalization of these processes, resulting in rapid improvement of serum-based fibrosis markers [23]. However, recent multicenter evidence indicates that metabolic dysfunction may adversely impact fibrosis regression after direct-acting antiviral therapy, even in patients achieving viral eradication [24]. In this context, persistent metabolic alterations after SVR—such as dyslipidemia—may modulate long-term hepatic remodeling. Together, these findings highlight the need for cautious interpretation of post-SVR improvements in non-invasive fibrosis markers and underscore the close interplay between metabolic health and liver disease evolution after HCV cure.

### 4.3. Clinical Implications

From a clinical perspective, these findings reinforce the importance of metabolic surveillance after HCV clearance. As viral eradication is increasingly achieved across diverse patient populations, long-term management must extend beyond virological outcomes to address metabolic and cardiovascular risk. Recent expert recommendations emphasize that post-SVR care should include systematic assessment of metabolic parameters, including lipid profiles, given their potential impact on long-term morbidity [25]. In this context, the sustained increases in total cholesterol and LDL-C observed in our cohort highlight the need for structured metabolic follow-up and individualized risk stratification in patients cured of chronic hepatitis C.

### 4.4. Study Limitations and Future Directions

Several limitations of this study should be acknowledged. First, this was a single-center observational study with a moderate sample size and incomplete follow-up at later time points, reflecting routine real-world clinical practice. Although linear mixed-effects models were used to account for missing data and repeated measurements, residual confounding cannot be fully excluded. The reduced sample size at extended follow-up reflects real-world clinical practice and may introduce a potential risk of selection bias; however, baseline characteristics were broadly comparable between groups.

Second, unmeasured lifestyle factors such as dietary habits, physical activity, alcohol consumption, and weight changes may have contributed to lipid variability following SVR. Information on lipid-lowering therapy was not systematically collected within the study protocol. However, treatment initiation following lipid increases would be expected to attenuate increases in LDL-C and cholesterol levels. Therefore, the sustained lipid elevations observed in our cohort are unlikely to be overestimated and may represent conservative estimates of post-SVR metabolic changes. Finally, the study was not designed to assess cardiovascular outcomes; therefore, the observed metabolic changes should not be interpreted as direct predictors of clinical events.

Despite these limitations, the real-world longitudinal design provides valuable insight into metabolic changes occurring after HCV cure beyond the early post-treatment period. Future studies in larger, multicenter cohorts with extended follow-up are warranted to better define the long-term cardiovascular implications of post-SVR lipid changes. Integrating metabolic, inflammatory, imaging, and clinical outcome data may further elucidate the mechanisms underlying post-cure metabolic remodeling and help establish evidence-based strategies for long-term monitoring and risk stratification in patients cured of chronic hepatitis C.

## 5. Conclusions

In this real-world study, cure of chronic hepatitis C with DAAs was associated with sustained changes in lipid metabolism. Total cholesterol and LDL-C increased after SVR and stabilized over time, independent of major changes in BMI or fibrosis stage. These findings underscore the importance of continued metabolic assessment after HCV cure, particularly in patients with additional cardiometabolic risk factors.

## Figures and Tables

**Table 1:** Baseline demographic and clinical characteristics of the study population.

Variable	Value
Number of patients, *n*	85
Age, years, mean ± SD	61.9 ± 11.8
Male n (%) Female n (%)	30 (35.3) 55 (64.7)
BMI, kg/m^2^, mean ± SD	26.57 ± 4.7
Cirrhosis (F4), n	31
Cardiometabolic comorbidity (%)	64.7%
Other comorbidity (%)	16.5%
Antiviral treatment regimen, n (%) Glecaprevir/pibrentasvir (GLE/PIB) Ledipasvir/sofosbuvir (LDV/SOF) Sofosbuvir/velpatasvir (SOF/VEL) Ombitasvir/paritaprevir/ritonavir + dasabuvir (OMB/PAR/RIT + DAS)	31 (36.5%) 30 (35.3%) 16 (18.8%) 8 (9.4%)
Total cholesterol, mg/dL, mean ± SD	157.7 ± 35.6
LDL-C, mg/dL, mean ± SD	94.6 ± 30.8
HDL-C, mg/dL, mean ± SD	51.6 ± 13.9
Triglycerides, mg/dL, median (IQR)	89 (73–118)
APRI score, median (IQR)	0.70 (0.50–1.20)
FIB-4 index, median (IQR)	2.20 (1.45–4.35)

**Table 2:** Baseline Characteristics of Patients With and Without SVR96 Follow-Up.

Variable	SVR96 Available	No SVR96 Follow-Up
Number of patients, n	23	62
Age, years (mean ± SD)	58.3 ± 13.7	63.2 ± 10.8
Sex (Male/Female)	7 (30.4%)/16 (69.6%)	23 (37.1%)/39 (62.9%)
BMI, kg/m^2^ (mean ± SD)	26.2 ± 2.7	26.7 ± 5.3
Cirrhosis (F4), n (%)	8 (34.8%)	23 (37.1%)
Cardiometabolic comorbidity, n (%)	11 (47.8%)	44 (71.0%)
Total cholesterol, mg/dL (mean ± SD)	163.9 ± 34.8	155.4 ± 35.9
LDL-C, mg/dL (mean ± SD)	99.6 ± 30.4	92.8 ± 31.0

**Table 3:** Longitudinal changes in non-invasive fibrosis indices after SVR.

Time Point	APRI, Median (IQR)	FIB-4, Median (IQR)	n
Baseline	0.70 (0.50–1.20)	2.20 (1.45–4.35)	85
SVR24	0.30 (0.20–0.40)	1.68 (1.13–2.65)	65
SVR48	0.30 (0.20–0.40)	1.52 (1.08–2.32)	52
SVR96	0.30 (0.20–0.40)	1.80 (0.94–2.70)	23

**Table 4:** Lipid parameters over time according to baseline fibrosis stage.

Time Point	Fibrosis Group	Total Cholesterol (mg/dL)	LDL-C (mg/dL)	HDL-C (mg/dL)	Triglycerides (mg/dL)	n
Baseline	F0–F2	166.1 ± 38.5	96.4 ± 32.1	56.6 ± 13.9	90 (69–117)	39
	F3–F4	150.6 ± 31.7	93.1 ± 30.0	47.3 ± 12.5	86 (74–117)	46
SVR24	F0–F2	185.9 ± 40.2	110.2 ± 34.4	57.8 ± 15.7	94 (80–113)	30
	F3–F4	173.9 ± 44.2	104.6 ± 32.2	50.6 ± 19.0	91 (73–124)	35
SVR48	F0–F2	192.5 ± 45.7	115.3 ± 41.3	56.9 ± 12.5	109 (91–132)	29
	F3–F4	184.6 ± 31.7	116.6 ± 28.6	53.1 ± 12.4	97 (83–125)	23
SVR96	F0–F2	175.3 ± 44.8	115.4 ± 46.3	48.1 ± 14.9	89 (74–115)	12
	F3–F4	183.6 ± 31.0	107.7 ± 29.8	51.8 ± 11.5	117 (99–125)	11

Values are presented as mean ± standard deviation for cholesterol fractions and median (interquartile range) for triglycerides. n indicates the number of patients with available paired lipid data at each time point.

**Table 5:** Lipid parameters over time according to cardiometabolic comorbidity status.

Time Point	Group	Total Cholesterol (mg/dL)	LDL-C (mg/dL)	n
Baseline	With cardiometabolic comorbidity	156.7 ± 37.8	92.9 ± 32.5	55
	Without cardiometabolic comorbidity	159.6 ± 31.7	97.8 ± 27.8	30
SVR24	With cardiometabolic comorbidity	177.7 ± 43.6	104.6 ± 33.5	46
	Without cardiometabolic comorbidity	183.8 ± 40.6	113.5 ± 32.2	19
SVR48	With cardiometabolic comorbidity	185.4 ± 39.1	112.7 ± 34.1	32
	Without cardiometabolic comorbidity	194.8 ± 41.5	120.9 ± 39.1	20
SVR96	With cardiometabolic comorbidity	181.1 ± 41.9	117.0 ± 48.2	11
	Without cardiometabolic comorbidity	177.6 ± 36.3	106.9 ± 28.6	12

Values are presented as mean ± standard deviation. n represents the number of patients with available paired lipid data at each time point. Analyses are descriptive.

## Data Availability

The dataset underlying this study contains sensitive patient information collected under conditions of confidentiality and pseudonymization, in accordance with the participants’ informed consent and the General Data Protection Regulation (EU 2016/679). Due to these ethical and legal restrictions, the full dataset cannot be made publicly available. De-identified data may be made available from the corresponding author upon reasonable request, subject to institutional and ethical approval.

## Abbreviations

The following abbreviations are used in this manuscript:

HCV	hepatitis C virus
DAA	direct-acting antiviral
SVR	sustained virological response
LDL-C	low-density lipoprotein cholesterol
HDL-C	high-density lipoprotein cholesterol
BMI	body mass index
APRI	aspartate aminotransferase-to-platelet ratio index
FIB-4	fibrosis-4 index
CI	confidence interval
GLE/PIB	glecaprevir/pibrentasvir
LDV/SOF	ledipasvir/sofosbuvir
OMB/PAR/RIT + DAS	ombitasvir/paritaprevir/ritonavir + dasabuvir
SOF/VEL	sofosbuvir/velpatasvir
HCC	hepatocellular carcinoma
